# Implantable collamer lens sizing based on measurement of the sulcus-to-sulcus distance in ultrasound biomicroscopy video clips and ZZ ICL formula

**DOI:** 10.1186/s12886-022-02583-9

**Published:** 2022-09-07

**Authors:** Jun Zhang, Jie Shao, Li Zheng, Xia Zhao, Shu Chen

**Affiliations:** Department of Ophthalmology, Hangzhou MSK Eye Hospital & MSK Eye Hospital of Hangzhou Medical College, Hangzhou, China

**Keywords:** Lenses, Intraocular, Phakic Intraocular Lens, Refractive Errors, Ultrasound Biomicroscopy

## Abstract

**Background:**

To evaluate a new method of implantable collamer lens (ICL) sizing based on ultrasound biomicroscopy (UBM) video clips.

**Methods:**

This observational study included consecutive patients with myopia and myopic astigmatism scheduled for V4c toric ICL (TICL) implantation (STAAR) at Hangzhou MSK Eye Hospital (October 2020 to November 2020). Sulcus-to-sulcus (STS) distance, lens thickness (LT), and clinical refraction were measured preoperatively. The ZZ ICL formula (provides the predicted vault height and refraction based on TICL size, intraocular meridian, power, and eye parameters, including STS distance and LT) was used to select TICL size and predict vault height and residual refraction, which was also compared with the STAAR software recommended. Vault and residual refraction were measured at 3 months postoperatively.

**Results:**

The analysis included 168 eyes in 84 patients. Postoperative vault size was comparable to that predicted by the ZZ ICL formula (528 ± 193 vs. 545 ± 156 μm, *P* = 0.227). Vault prediction error (PE) by the ZZ ICL formula was within 100, 300, and 500 μm in 40.48%, 88.10%, and 100% of eyes, respectively. Spherical equivalent (SE) and absolute cylindrical refractive error were 0.36 ± 0.48 and 0.40 ± 0.31 D at 3 months postoperatively. The SE PE, absolute cylindrical PE, and percentages of eyes with an absolute cylindrical PE within ± 0.50 D and ± 1.00 D were lower for the ZZ ICL formula than for the STAAR software (*P* < 0.01).

**Conclusions:**

Combining measurements obtained in UBM video clips with the ZZ ICL formula provides an effective method of sizing TICLs and predicting vault height and residual refractive error.

**Supplementary Information:**

The online version contains supplementary material available at 10.1186/s12886-022-02583-9.

## Background

The implantable collamer lens (ICL) is a phakic intraocular lens implanted in the posterior chamber of the eye. ICLs have proven safe and effective for the correction of a wide range of refractive errors [1]. Parameters used to assess the success of ICL surgery include the presence/absence of unexpected refractive error and the size of the vault, which is defined as the distance between the posterior ICL surface and the anterior crystalline lens surface [2, 3]. Repeat surgery to adjust the vault, either by ICL rotation or lens exchange, is needed in approximately 0.8% of cases [4].

Reducing the risk of an abnormal vault requires accurate sizing of the ICL before surgery. The most commonly used sizing method is based on measurements of the horizontal corneal white-to-white (WTW) distance and anterior chamber depth (ACD). An alternative technique utilizes ultrasound biomicroscopy (UBM) to measure the ciliary sulcus-to-sulcus (STS) distance. The UBM-based method is founded on the concept that the ICL is designed to be positioned along one diameter of the annular ciliary sulcus, whereas the conventional method based on the WTW distance may have limitations because the correlation between the ciliary STS distance and WTW diameter is poor [5, 6]. Some studies have incorporated direct measurements of the internal structures of the ciliary STS distance into their formulae for preoperative sizing [7–9]. Although a meta-analysis identified no significant differences in vault size between WTW-based and STS distance-based sizing methods [4], this finding is not consistent with our clinical experience. Despite the theoretical advantages of the STS distance-based approach, the accuracy of this method is affected by subjective factors and hence operator experience.

Therefore, a video clip obtained by high-frequency UBM would provide a more comprehensive evaluation of ciliary sulcus morphology and a more intuitive frame-by-frame comparison measurement with measured trace than a single static image and thereby help even lesser qualified technicians optimize the measurement of the maximal STS distance. The maximal STS distance in the direction of the ICL long axis should theoretically be the effective STS distance and better suited for estimation of the ICL chord height. The vault height can then be estimated from the chord height [10]. Subsequently, we developed the Zhang & Zheng ICL (ZZ ICL) formula (available at www.zzcal.com) to estimate the postoperative vault based on the horizontal and vertical maximal STS distances, the expected direction of the ICL, and the crystalline LT.

In order to reduce the influence of unexpected refractive error, the optimization of refraction should include compensation for both effective lens position (ELP) [11, 12] and surgery-induced astigmatism (SIA) [13, 14], which affect the postoperative spherical equivalent (SE) and astigmatism, respectively. We routinely utilize the ICL manufactured by STAAR Surgical, and although the formula provided by the STAAR website for the calculation of ICL power has not been disclosed because it is proprietary, it does not take ELP or SIA into account. The ELP can be calculated from an estimation of vault height, and the determination of SIA has been described before [13, 15]. Therefore, we have modified the ZZ ICL formula to include ELP and SIA with the aim of optimizing refractive outcomes.

The first aim of the present study was to develop an effective method of determining STS distance using UBM-derived video clips. The second aim was to evaluate the accuracy of the modified ZZ ICL formula in the estimation of vault height. The third aim was to compare the residual refraction prediction error (PE) between the ZZ ICL formula and the software provided on the STAAR website.

## Methods

### Study design and patients

This observational study included consecutive patients scheduled for toric ICL (TICL) surgery at Hangzhou MSK Eye Hospital between October 2020 and November 2020. The inclusion criteria were (1) aged 21–55 years old; (2) myopia between -3.00 diopter sphere (DS) and -20.00 DS; (3) refractive astigmatism between 0.00 diopter cylinder (DC) and -5.00 DC; (4) meeting the indications for TICL implantation; (5) scheduled for implantation of a V4c TICL (STAAR Surgery, Nidau, Switzerland) using KS-aquaPORT technology. The exclusion criteria were (1) keratoconus, crystalline lens deformity, crystalline lens heterotopia, glaucoma, cataract, uveitis, retinal detachment, or macular degeneration; (2) previous ocular or intraocular surgery; (3) contraindications to TICL implantation surgery. In addition, cases with a follow-up period < 3 months were excluded from the final analysis.

Institutional review board approval was obtained from the medical research ethics committee of Hangzhou MSK Eye Hospital (approval number: MSKLL20201006). All procedures in this study adhered to the tenets of the Declaration of Helsinki. All participants were informed about the risks and benefits of the procedure and provided written informed consent. This study is registered in the Chinese Clinical Trials Registry (registration number: ChiCTR 2,000,038,862).

### Preoperative and postoperative examinations

All patients underwent a complete ophthalmic examination. Before the examination, each patient was instructed to avoid using visual display terminals or reading books for at least 3 h [16]. The measurements were carried out in a daily illumination/interpupillary environment (4 lx).

WTW distance was determined using anterior segment tomography (Sirius; CSO, Florence, Italy). Horizontal and vertical STS distances were obtained using video clips acquired by high-frequency B-scan diagnostic UBM (AVISO, Quantel Medical, Clermont-Ferrand, France). The crystalline LT was estimated using optical biometry (IOLMaster 700; Carl Zeiss, Jena, Germany). The vault height was measured manually using the built-in caliper tool of the OCT system (OCT; Cirrus HD-OCT 5000; Carl Zeiss, Jena, Germany). All measurements were taken from the highest point of the central region. Three readings were taken for each parameter, and the average value was used for the analysis.

### Measurement of STS distance by UBM

All UBM-based measurements were made by the same examiner with six years of experience. A probe with a 50-MHz transducer (Axial resolution: 35 μm, Lateral resolution: 60 μm) was used for horizontal and vertical STS distance measurements. After finding the image with four high-reflection bands (representing the central parts of the anterior and posterior surfaces of the cornea and crystalline lens; Supplementary Fig. 1), the probe was rotated clockwise/counterclockwise about 10º along the measuring axis to obtain images on either side of the image with the four high-reflection bands (Supplementary Fig. 2). Two video clips (10 s, 100 frames) were collected for each meridian. Measurements were made using the built-in linear caliper with the average velocity set at 1550 m/s (the default built-in device’s parameter). STS distance on each meridian was recorded for a clear view of the largest possible STS distance within 10 × 2 s cine length.

### Calculation of the refraction PE

The SE PE for each formula was calculated as:

$${\text{SE PE}}_{\text{ZZ ICL}}={\text{predicted SE}}_{\text{ZZ ICL}}- {\text{SE}}_{\text{POST}}$$

$${\text{SE PE}}_{\text{STAAR}}={\text{predicted SE}}_{\text{STAAR}}- {\text{SE}}_{\text{POST}}$$

where SE_POST_ represents the residual SE refraction at 3 months postoperatively, predicted SE_ZZ ICL_ and predicted SE_STAAR_ represent the predicted residual refraction for the respective formula, and SE PE_ZZ ICL_ and SE PE_STAAR_ represent the SE PE for the corresponding formula. The vector analysis method described by Alpins [17] was used to calculate the absolute and vector cylindrical PE for each formula similarly.

### TICL implantation

All patients were implanted with a V4c TICL (STAAR Surgical, Nidau, Switzerland). The TICL size (12.1, 12.6, 13.2, or 13.7 mm), intraocular meridian, and power were determined according to the ZZ ICL formula (freely available at www.zzcal.com). The predicted vault height and predicted refraction were returned after TICL size, intraocular meridian, power, and eye parameters were input into the ZZ ICL formula (Supplementary Fig. 3). The calculation of predicted vault height involved the following four steps: (1) calculation of the STS distance of the target meridian using the horizontal and vertical STS distance (Supplementary Fig. 4a); the ciliary ring was regarded as elliptical, and the average velocity was converted from 1550 m/s to 1586.5 m/s; (2) calculation of the chord height of the TICL (defined as the distance from the inner surface of the lens to the plane of the four haptic loops of the lens) using the STS distance and planned TICL diameter (Supplementary Fig. 4b); (3) calculation of the predicted vault height by subtracting both the percentage of crystalline LT and the fixed value for crystalline lens forward movement [10] from the chord height (Supplementary Fig. 4c); (4) if the calculated vault height did not match clinical expectations, steps 1–3 were repeated after changing the planned intraocular meridian or size. In this study, the default SIA was 0.20 D @ 0º based on previous clinical results (Supplementary Fig. 5). The TICL power was adjusted to be consistent with the planned implantation.

Surgery was performed using a 3-mm clear temporal corneal incision. The TICL was inserted using an injector cartridge (STAAR Surgical) and rotated to the target position and meridian. Correct positioning of the TICL in the center of the pupillary zone was achieved with the help of an image-guidance system (Callisto Eye; Carl Zeiss AG, Dublin, CA, USA).

### Data collection

All data obtained by the preoperative and postoperative examinations were collected. TICL size, intraocular meridian, power, predicted vault height and predicted residual refraction determined by the ZZ ICL formula were recorded. TICL size, power, and predicted residual refraction provided by the STAAR website software were also recorded. Additionally, vault height and refraction data were collected at 3 months postoperatively. Vault PE was the absolute value of the difference between the actual vault height measured after surgery and the vault height expected by the ZZ ICL formula. Vault PE =|ZZ ICL predicted vault—achieved vault|.

### Statistical analysis

All parameters except for absolute cylindrical PE were normally distributed. The paired t-test and Wilcoxon signed-rank test were used to compare normally-distributed variables and non-normally distributed variables, respectively. The chi-squared test was used to analyze the frequency of absolute cylindrical PE within 0.50 D, and within 1.00 D. The asymptotic P-value of the McNemar test was recorded since the number of observations was sufficient for both comparisons. All analyses were performed using SPSS 19.0 (IBM, Armonk, NY, USA). Two-sided *P* < 0.05 was considered statistically significant.

## Results

### Characteristics of the study participants

Among 168 eyes in 84 patients were included in the final analysis (Supplementary Fig. 6). The preoperative clinical characteristics are presented in Supplementary Table 1.

### Recommended TICL size

**Fig. 1:**
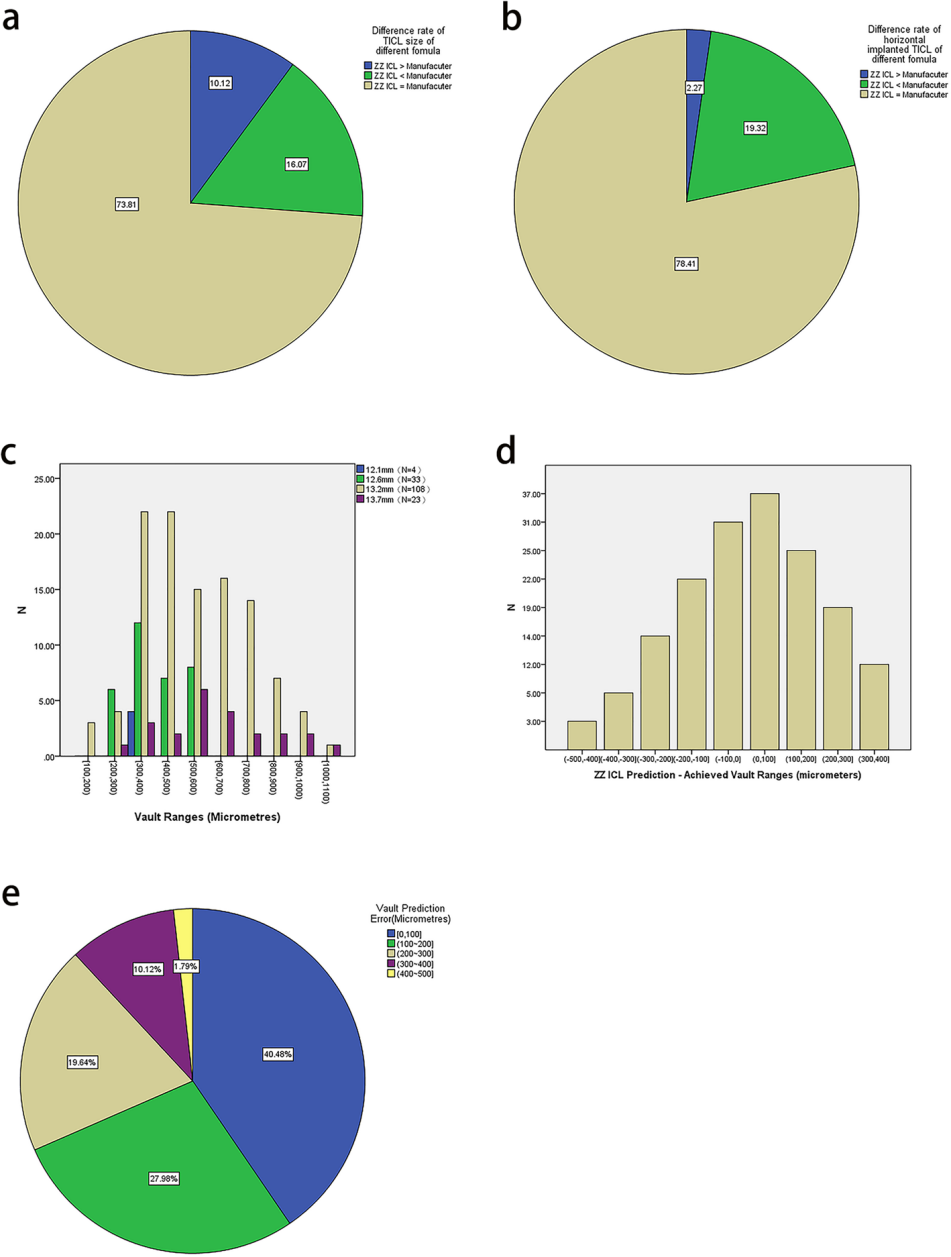
**a** Distribution of the percentage difference in toric implantable collamer lens (TICL) size between the Zhang & Zheng implantable collamer lens (ZZ ICL) formula and the STAAR formula. **b** Distribution of the percentage difference in TICL size between the two formulae in patients with a horizontally implanted TICL. **c** Distribution of the vault ranges for TICLs of differing sizes. **d** Distribution of the difference in vault size between the achieved value and the value predicted by the ZZ ICL formula. **e** Distribution of the vault prediction error for the ZZ ICL formula

All eyes in all patients were implanted with a TICL that was sized according to the ZZ ICL formula (Fig. 1a and Supplementary Table 2). Since the TICL size recommended by the manufacturer does not consider the vertical position, we performed an additional comparison excluding the TICLs implanted with a vertical orientation (Fig. 1b) [18, 19].

### Comparison of predicted and postoperative vault size

The vault parameters are summarized in Table 1 and Fig. 1c–e. Postoperative vault size was not significantly different from that predicted by the ZZ ICL formula for all eyes (528 ± 193 vs. 545 ± 156 μm, *P* = 0.227) for eyes implanted with a smaller TICL than that recommended by the STAAR software (*P* = 0.969), and for eyes implanted with a TICL of the same size as that suggested by STAAR (*P* = 0.648). However, postoperative vault size was significantly smaller than that predicted by the ZZ ICL formula for eyes implanted with a larger TICL than that recommended by STAAR (*P* = 0.014), raising the possibility that some of these patients might have worse outcomes. The numbers of eyes with a vault PE within 100, 300, and 500 μm were 68 (40.48%), 148 (88.10%), and 168 (100%), respectively.

**Table 1:** Vault characteristics

Parameter	n	Achieved postoperatively	Predicted by ZZ ICL	P
Vault height (µm)	168	528 ± 193 (160 − 1040)	545 ± 156 (200 − 1186)	0.227
Larger size implanted^a^	17	404 ± 119 (240 − 650)	513 ± 111 (316 − 690)	0.014
Smaller size implanted^a^	27	505 ± 189 (280 − 1040)	506 ± 157 (258 − 830)	0.969
Same size implanted^a^	124	550 ± 196 (160 − 1040)	558 ± 160 (200 − 1186)	0.648

Data are shown as mean ± SD (range). ZZ ICL: Zhang & Zheng implantable collamer lens

^a^In comparison to the size recommended by the STAAR website software

### Residual refraction

**Fig. 2:**
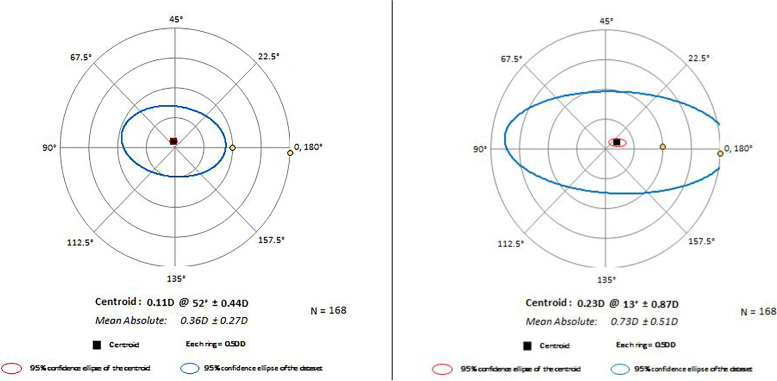
The absolute and vector cylindrical refraction prediction error for each formula

The SE and absolute cylindrical refractive error were 0.36 ± 0.48 D and 0.40 ± 0.31 D at 3 months postoperatively. The refractive PE parameters for the ZZ ICL formula and STAAR software are summarized in Table 2, Supplementary Table 3, and Fig. 2. The SE PE and absolute cylindrical PE were both significantly smaller for the ZZ ICL formula than for the STAAR website software (*P* < 0.01; Table 2). The percentages of eyes with an absolute cylindrical PE within ± 0.50 D and ± 1.00 D were significantly lower for the ZZ ICL formula than for the STAAR software (*P* < 0.01; Supplementary Table 3). The vector cylindrical PE was also noticeably smaller for the ZZ ICL formula than for the STAAR software (Fig. 2).

**Table 2:** Refractive prediction errors

Parameter (mean ± SD)	ZZ ICL	STAAR	P
Spherical equivalent prediction error (D)	0.01 ± 0.48	0.16 ± 0.48	< 0.001
Absolute cylindrical prediction error (D)	0.40 ± 0.27	0.82 ± 0.54	< 0.001

Data are shown as mean ± SD

*ZZ ICL* Zhang & Zheng implantable collamer lens

## Discussion

The objectives of the present study were to compare the performances of the ZZ ICL formula and the STAAR website software in the prediction of TICL size, vault height, and residual refraction error. A notable finding was that the size of the implanted TICL (as recommended by the ZZ ICL formula) was larger than that recommended by the STAAR software in 10.1% of eyes and smaller than that recommended by STAAR in 16.1% of eyes. Furthermore, the postoperative vault size was not significantly different from that predicted by the ZZ ICL formula. The SE PE and absolute cylindrical PE were significantly smaller for the ZZ ICL formula than when using the STAAR software. Taken together, the present study suggests that UBM and the ZZ ICL formula can improve the selection of TICL size and estimation of vault height compared with the software provided by the manufacturer of the TICL.

Selecting an appropriate ICL size and achieving a safe vault size are critical to minimizing the risks of complications after ICL implantation [4, 20]. Still, one of the main challenges is the prediction of vault height [21, 22]. The usual approach to sizing the ICL involves a measurement of the horizontal WTW and ACD followed by the use of the manufacturer’s software, but approximately 20% of eyes fall outside the accepted vault range [23, 24]. An ICL is designed to be positioned along one diameter of the annular ciliary sulcus, but the correlation between the ciliary STS distance and WTW diameter is poor [5], suggesting that STS distance-based sizing might be more appropriate. Therefore, this study investigated whether the UBM-based measurement of the STS distance and the use of the ZZ ICL formula might provide a better alternative to the STAAR software. This study showed that the TICL size recommended by the ZZ ICL formula was larger than that suggested by STAAR in 10.1% of eyes and smaller than that advised by STAAR in 16.1% of eyes. These results are similar to those of Kojima et al. [25]; 13.9% showed low vault, and 13.9% showed high vault through traditional ICL sizing methods. In addition, the results in the horizontally TICL group were similar to Reinstein et al. [22]; 6% showed low vault, and 34% showed high vault. Therefore, these results indirectly support the clinical significance of the ZZ ICL formula. Importantly, the vault height predicted by the ZZ ICL formula was not significantly different from the postoperative vault height. We suggest that UBM-based measurement of sulcus diameter and use of the ZZ ICL formula might be a better approach to selecting TICL size.

Although a meta-analysis found no significant differences in vault size between WTW-based and STS distance-based sizing methods [4], this finding might have been influenced by inaccurate measurement of the STS distance and by heterogeneity among the included studies. One possible factor is operator inexperience. Another possible factor is the use of a single static image showing four high-reflection bands. The STS distance in this section often does not match the maximal STS distance of the target direction (the effective STS distance). Therefore, this study used an UBM-derived video clip acquired by simple rotation of the probe to obtain a more comprehensive evaluation of the ciliary sulcus morphology. Theoretically, a highly experienced UBM technician might obtain a similar STS distance using UBM-derived static images, but it is easier to help even lesser qualified technicians intuitively identify the maximal STS distance by comparing the measured trace of one frame with its previous and subsequent frames. Furthermore, this study took into account that the STS distance measurement path traverses approximately equal distances along with the aqueous humor and crystalline lens. Therefore, rather than using an average velocity of 1550 m/s, a setting of 1586.5 m/s was used, which is the average velocity for the aqueous humor and crystalline lens. In addition, the percentage of the LT was used to estimate the part above the plane of the TICL distal haptics.

**Fig. 3:**
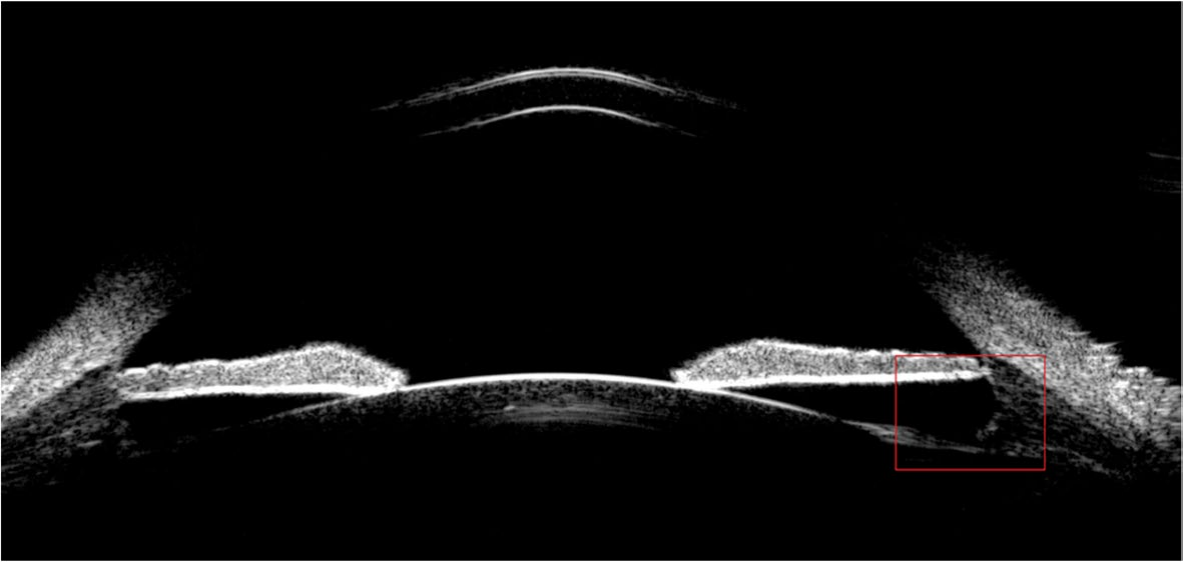
Ultrasound biomicroscopy image showing a wide ciliary sulcus

The present study only included patients scheduled for TICL implantation to facilitate evaluations of the intraocular orientation of the TICL and the correction of astigmatic refraction. No spontaneous rotation exceeding 10º occurred during the 3-month follow-up period. It also indirectly suggests that the actual landing zone of the TICL haptics is another factor contributing to vault prediction error [23]. In this study, patients with an error > 400 µm appeared to have a widened ciliary sulcus shape, as shown in Fig. 3. The frequencies of different landing zone types have been reported previously [26, 27]. Although there is currently no reliable method to reduce this kind of error, it could be anticipated that the present study might facilitate the development of improved techniques to estimate vault size.

Optimizing SE and astigmatism are critical to reducing the incidence of unexpected refractive error. Although the STAAR website formula achieves good refractive correction outcomes, it can be hypothesized that the outcomes might be further optimized through the use of the ZZ ICL formula with compensation for both the ELP [11, 12] and SIA [13, 14]. Indeed, the SE PE and absolute cylindrical PE were significantly smaller for the ZZ ICL formula than for the STAAR software. In addition, the ZZ ICL formula achieved higher proportions of cases with an absolute cylindrical PE within ± 0.50 D and ± 1.00 D. An advantage of the ZZ ICL formula was also evident in the statistical analysis of the vector cylindrical refraction PE. A reduction in the standard deviation of the cylindrical refraction PE should be of particular clinical significance.

Since the STAAR formula is proprietary and the manufacturer’s standard calculation is not disclosed, the possible reasons are as follows. 1) When the vault height is changed, the diopter calculated by the STAAR method does not change. ZZ ICL will affect the diopter calculation result according to the different input vault heights. 2) The STAAR method does not require SIA. ZZ ICL will calculate different diopter results according to the input SIA, achieving a better treatment personalization.

Our study has several limitations. First, the sample size was quite small, so a larger study will be needed to confirm our findings. Second, although the measurement method of the STS distance has been less affected by subjective factors, our study was carried out at a single center. Therefore, some degree of selection bias and information bias cannot be ruled out. Third, the UBM results may be subjective, and ensuring the central measurement may be very difficult in a clinical setting. Fourth, only one surgeon performed all the procedures, which introduces selection bias. Fifth, the measurements were carried out in a daily illumination/interpupil environment, which could introduce variation. Sixth, instantaneous vault height was obtained, and this may have introduced information bias. Indeed, vault dynamism is an important component of ICL formulas [28]. Finally, the study follow-up was limited to 3 months, so longer-term outcomes could not be evaluated. Further investigations are needed to elucidate whether there are long-term vault changes.

## Conclusions

In conclusion, video clips obtained by UBM can facilitate the measurement of STS distance. Furthermore, the ZZ ICL formula is useful for ICL sizing and the prediction of postoperative vault height. Additionally, the ZZ ICL formula was better at predicting residual refraction than the STAAR website formula. We anticipate that the use of UBM-acquired video clips and the ZZ ICL formula may help to optimize the clinical outcomes of ICL.

## Supplementary Information

**Additional file 1: Supplementary Figure 1.** Representative ultrasound biomicroscopy image showing the four high-reflection bands representing the central parts of the anterior and posterior surfaces of the cornea and crystalline lens.

**Additional file 2: Supplementary Figure 2.** Schematic diagram illustrating the acquisition of sections on either side of the image with four high-reflection bands.

**Additional file 3: Supplementary Figure 3.** Screenshot illustrating the use of the Zhang & Zheng implantable collamer lens (ZZ ICL) formula.

**Additional file 4: Supplementary Figure 4.** **a** The thick black ellipse simulates the ciliary ring, the two black diameters simulate the horizontal and vertical sulcus-to-sulcus (STS) distance, and the brown line simulates the planned intraocular meridian of the toric implantable collamer lens (TICL). **b** The thick black arc simulates the TICL, the thin black line simulates the actual STS distance, and the brown line simulates chord height. **c** The thick black arc simulates the TICL, the thick black ellipse simulates the crystalline lens, the thin black line simulates the actual STS distance, and the brown line simulates vault height.

**Additional file 5: Supplementary Figure 5.** Statistical analysis of surgery-induced astigmatism (SIA) of Jun Zhang at 3 months postoperatively (50 cases, 20–30 years old; 3 × 1.6 mm temporal arc incision made at the corneoscleral margin).

**Additional file 6: Supplementary Figure 6.** Flowchart showing enrolment of the study participants.

**Additional file 7: Supplementary Table 1.** Preoperative clinical characteristics of the study participants. **Supplementary Table 2.** Size and intraocular orientation of the toric implantable collamer lens. **Supplementary Table 3.** The number and percentage of eyes with an absolute cylindrical prediction error within ±0.50 D and ±1.00 D.

## Data Availability

All data generated or analyzed during this study are included in this published article [and its supplementary information files].

## Abbreviations

ACD: Anterior chamber depth
DC: Diopter cylinder
DS: Diopter sphere
ELP: Effective lens position
ICL: Implantable collamer lens
LT: Lens thickness
PE: Prediction error
SE: Spherical equivalent
SIA: Surgery-induced astigmatism
STS: Sulcus-to-sulcus
TICL: Toric ICL
UBM: Ultrasound biomicroscopy
WTW: White-to-white
ZZ ICL: Zhang & Zheng ICL
