# Supplementary figures and images for: Implantable collamer lens sizing based on measurement of the sulcus-to-sulcus distance in ultrasound biomicroscopy video clips and ZZ ICL formula
Source: BMC Ophthalmol. 2022 Sep 7;22:363. doi: 10.1186/s12886-022-02583-9 (PMC9454160; doi:10.1186/s12886-022-02583-9)

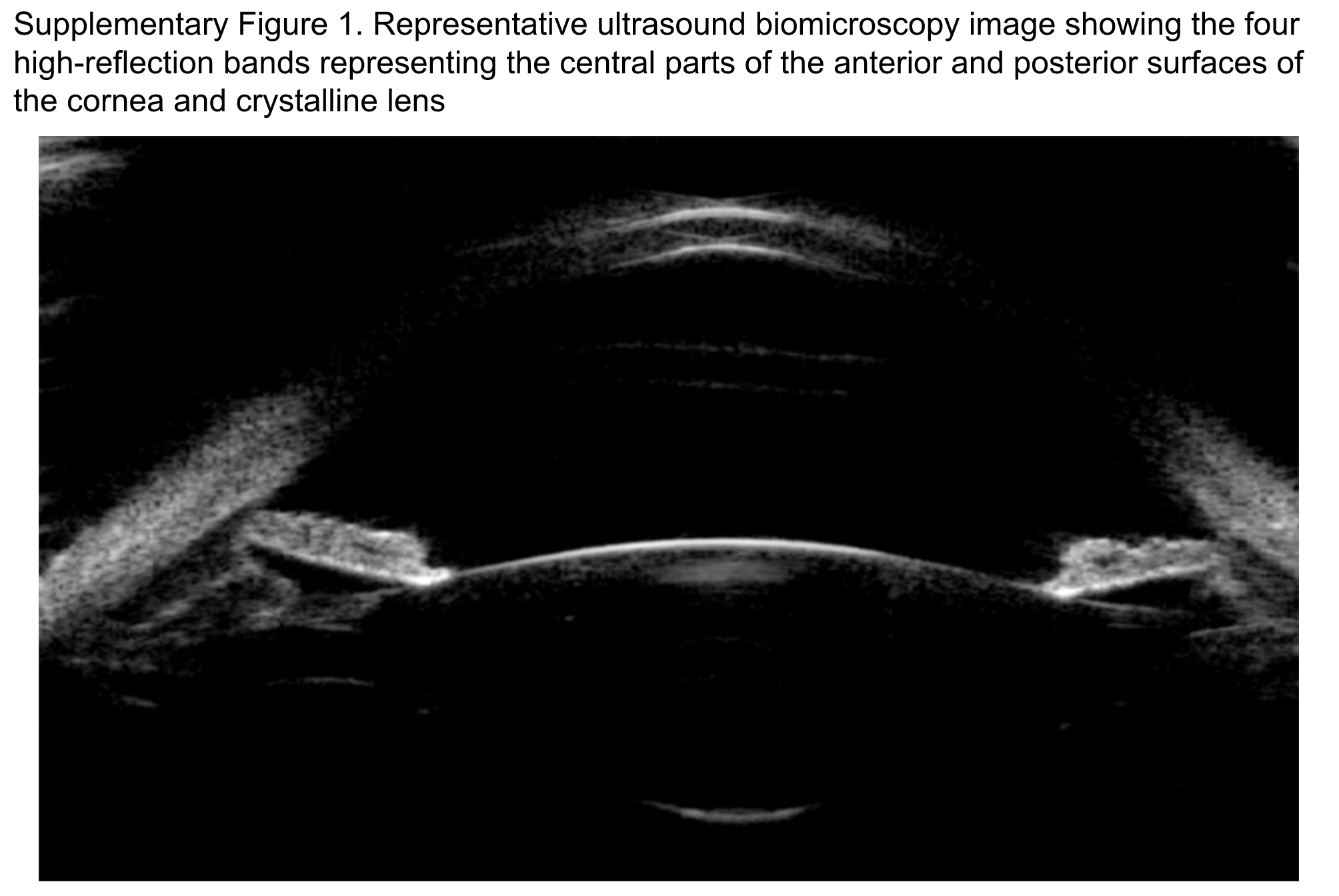

Supplement: Supplementary file 1 — Additional file 1: Supplementary Figure 1. Representative ultrasound biomicroscopy image showing the four high-reflection bands representing the central parts of the anterior and posterior surfaces of the cornea and crystalline lens. [file 12886_2022_2583_MOESM1_ESM.jpg]

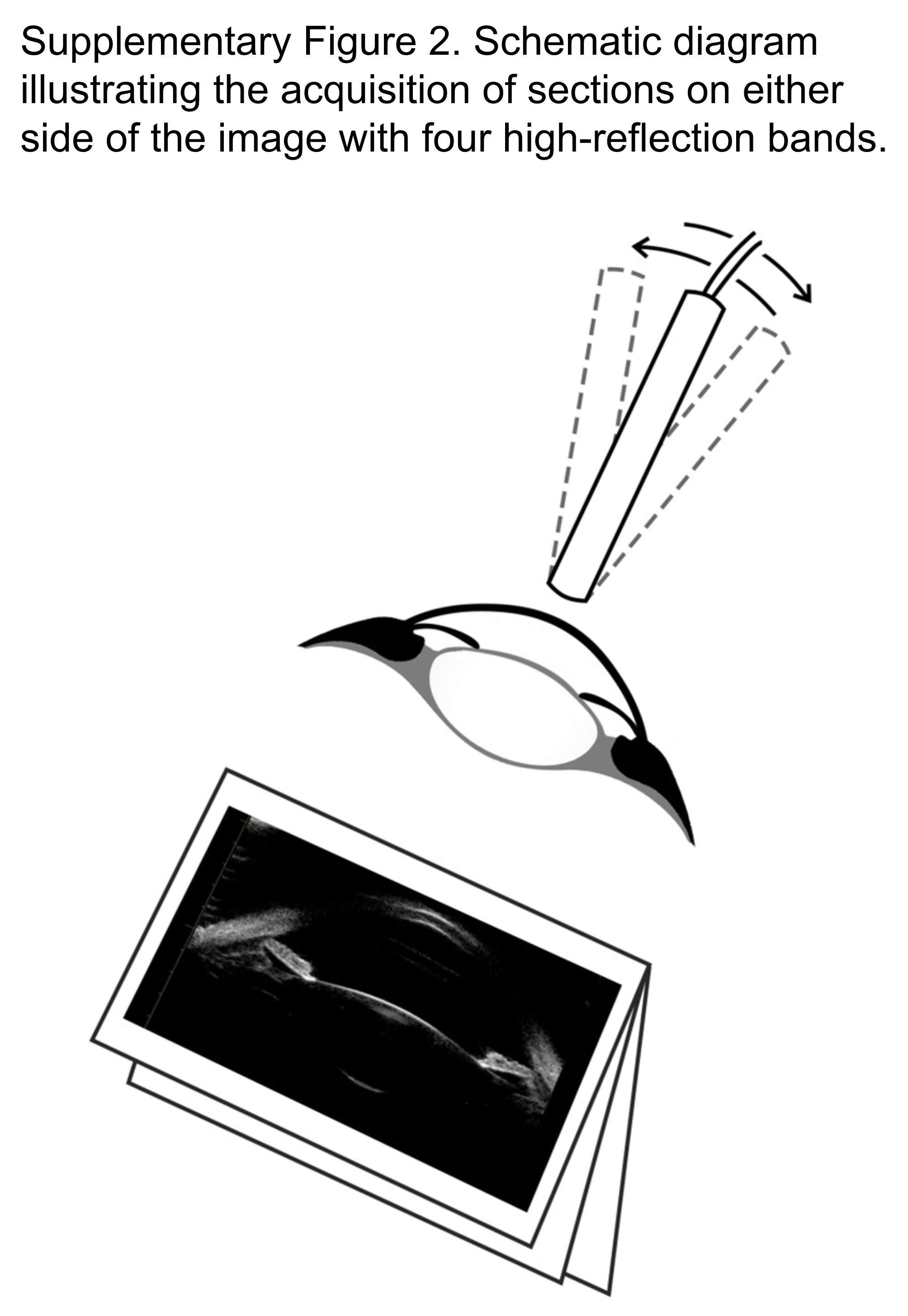

Supplement: Supplementary file 2 — Additional file 2: Supplementary Figure 2. Schematic diagram illustrating the acquisition of sections on either side of the image with four high-reflection bands. [file 12886_2022_2583_MOESM2_ESM.jpg]

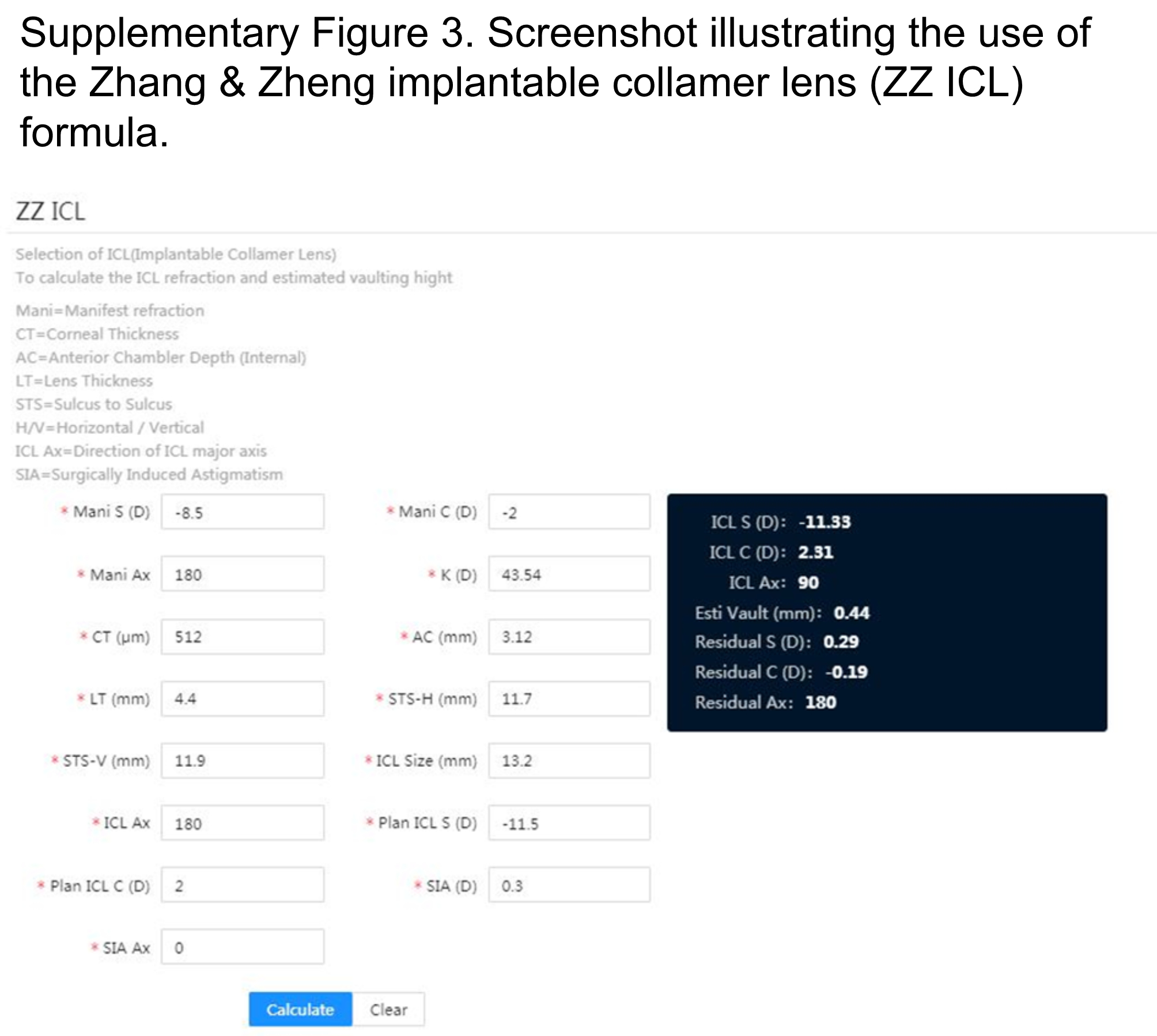

Supplement: Supplementary file 3 — Additional file 3: Supplementary Figure 3. Screenshot illustrating the use of the Zhang & Zheng implantable collamer lens (ZZ ICL) formula. [file 12886_2022_2583_MOESM3_ESM.jpg]

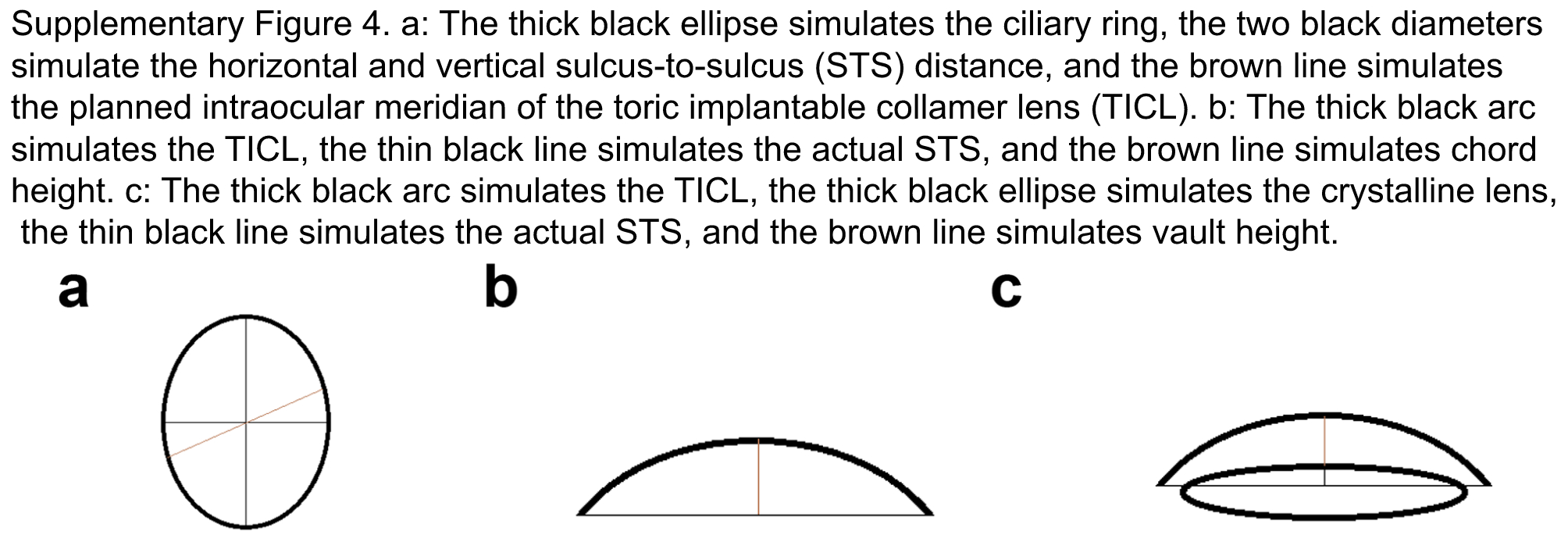

Supplement: Supplementary file 4 — Additional file 4: Supplementary Figure 4. a The thick black ellipse simulates the ciliary ring, the two black diameters simulate the horizontal and vertical sulcus-to-sulcus (STS) distance, and the brown line simulates the planned intraocular meridian of the toric implantable collamer lens (TICL). b The thick black arc simulates the TICL, the thin black line simulates the actual STS distance, and the brown line simulates chord height. c The thick black arc simulates the TICL, the thick black ellipse simulates the crystalline lens, the thin black line simulates the actual STS distance, and the brown line simulates vault height. [file 12886_2022_2583_MOESM4_ESM.jpg]

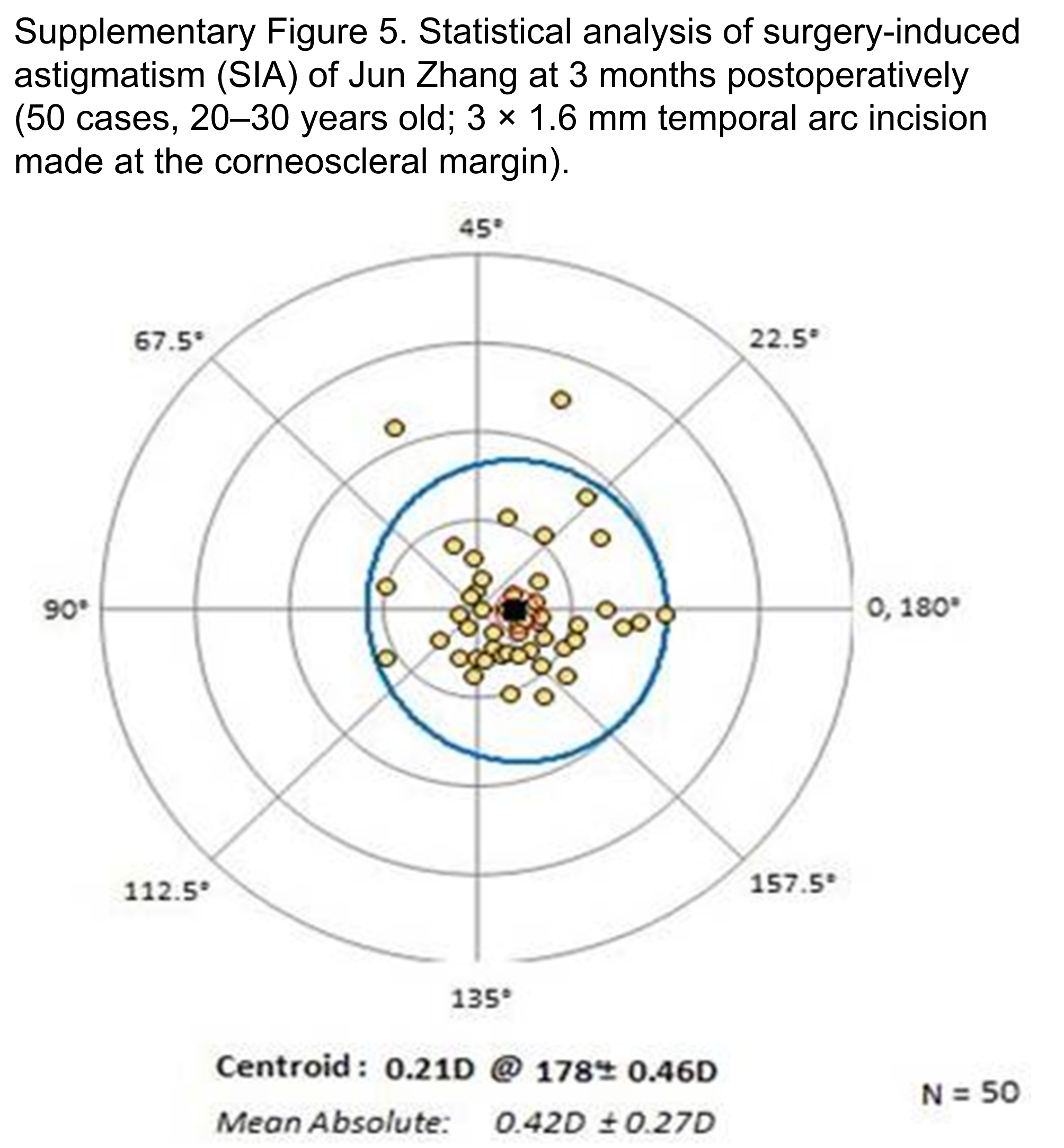

Supplement: Supplementary file 5 — Additional file 5: Supplementary Figure 5. Statistical analysis of surgery-induced astigmatism (SIA) of Jun Zhang at 3 months postoperatively (50 cases, 20–30 years old; 3 × 1.6 mm temporal arc incision made at the corneoscleral margin). [file 12886_2022_2583_MOESM5_ESM.jpg]

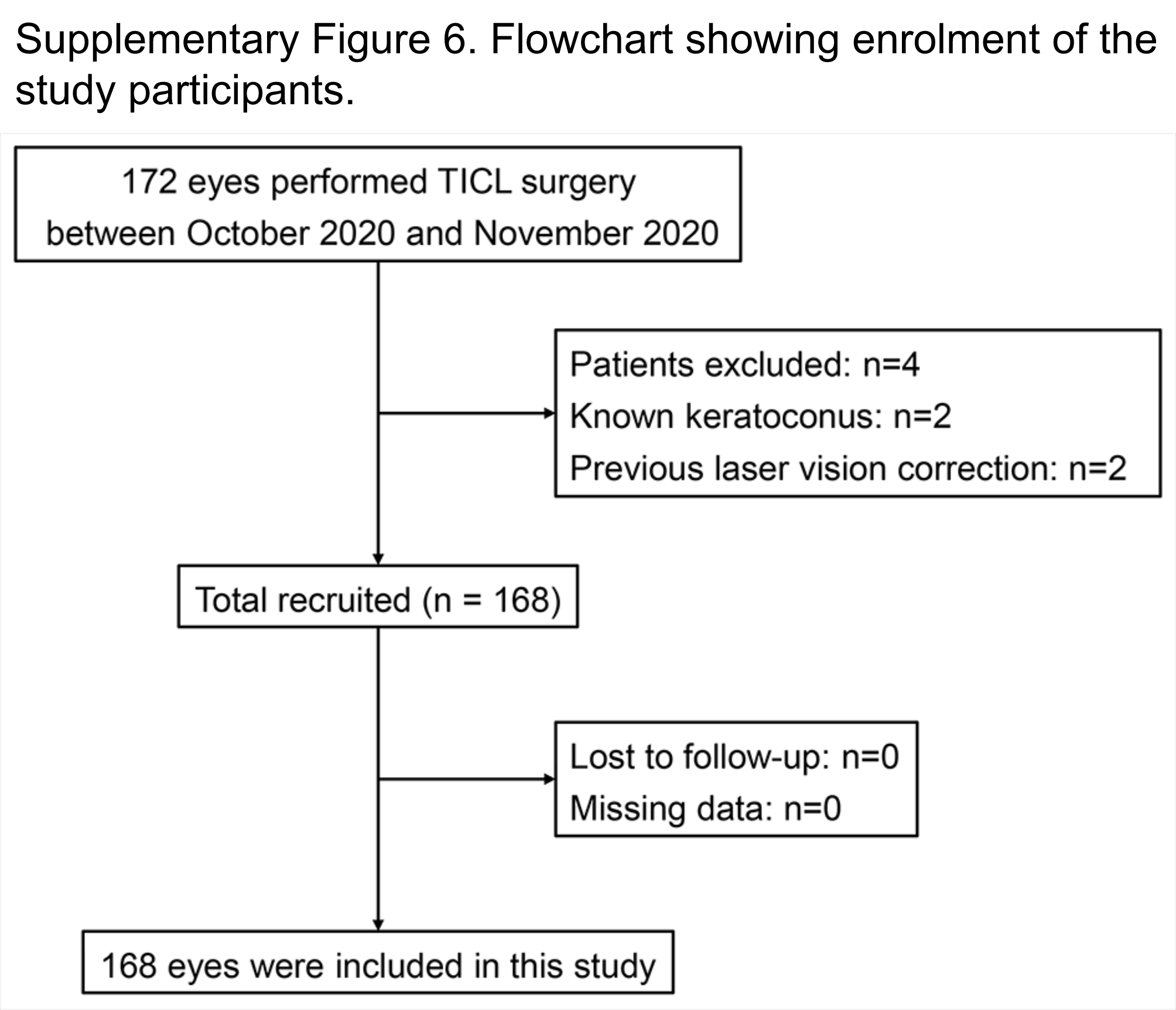

Supplement: Supplementary file 6 — Additional file 6: Supplementary Figure 6. Flowchart showing enrolment of the study participants. [file 12886_2022_2583_MOESM6_ESM.jpg]
