# Supplementary material for: Implantable collamer lens sizing based on measurement of the sulcus-to-sulcus distance in ultrasound biomicroscopy video clips and ZZ ICL formula
Source: BMC Ophthalmol. 2022 Sep 7;22:363. doi: 10.1186/s12886-022-02583-9 (PMC9454160; doi:10.1186/s12886-022-02583-9)
Supplement: Supplementary file 7 — Additional file 7: Supplementary Table 1. Preoperative clinical characteristics of the study participants. Supplementary Table 2. Size and intraocular orientation of the toric implantable collamer lens. Supplementary Table 3. The number and percentage of eyes with an absolute cylindrical prediction error within ±0.50 D and ±1.00 D. [file 12886_2022_2583_MOESM7_ESM.docx]

**Supplementary Table 1. Preoperative clinical characteristics of the study participants**

| **Variable** | **N** | **Value (mean±SD or %)** | **Range** |
| --- | --- | --- | --- |
| Age (years) | 84 | 24.58±4.18 | 21−35 |
| Sex (male) | 84 | 29.76% | - |
| Manifest refraction |  |  |  |
| Spherical (D) | 168 | -8.14±2.20 | -14.75−-2.75 |
| Cylindrical (D) | 168 | -1.97±0.87 | -4.50−-0.50 |
| Intraocular pressure (mmHg) | 168 | 14.24±2.49 | 7.30−20.30 |
| Central corneal thickness (mm) | 168 | 509.47±30.02 | 418−582 |
| Anterior chamber depth (mm) | 168 | 3.31±0.23 | 2.80−3.86 |
| Lens thickness (mm) | 168 | 3.58±0.22 | 3.04−4.15 |
| Axial length (mm) | 168 | 27.01±1.21 | 23.33−29.89 |
| White-to-white distance (mm) | 168 | 11.96±0.35 | 11.19−12.80 |
| Sulcus-to-sulcus distance (mm) |  |  |  |
| Horizontal | 168 | 11.68±0.39 | 10.31−12.65 |
| Vertical | 168 | 11.98±0.37 | 10.60−12.89 |

**Supplementary Table 2. Size and intraocular orientation of the toric implantable collamer lens**

| **Variable** | **n** | **%** |
| --- | --- | --- |
| TICL size recommended by the STAAR software (mm) | | |
| 12.1 | 0 | 0.00% |
| 12.6 | 33 | 19.64% |
| 13.2 | 110 | 65.48% |
| 13.7 | 25 | 14.88% |
| TICL size recommended by the ZZ ICL formula (mm) | | |
| 12.1 | 4 | 2.38% |
| 12.6 | 33 | 19.64% |
| 13.2 | 108 | 64.29% |
| 13.7 | 23 | 13.69% |
| TICL orientation recommended by the ZZ ICL formula | | |
| Horizontal (0º±30º) | 88 | 52.38% |
| Vertical (90º±30º) | 80 | 47.62% |

TICL: toric implantable collamer lens; ZZ ICL: Zhang & Zheng implantable collamer lens.

**Supplementary Table 3.** **The number and percentage of eyes with an absolute cylindrical prediction error within ±0.50 D and ±1.00 D**

| **Parameter** | **ZZ ICL** | **STAAR** | **P** |
| --- | --- | --- | --- |
| ±0.50 D | 111 (66.1%) | 60 (35.7%) | <0.001 |
| ±1.00 D | 163 (97.0%) | 122 (72.6%) | <0.001 |

Data are shown as n (%). ZZ ICL: Zhang & Zheng implantable collamer lens.
